# Genetic analysis of ALS cases in the isolated island population of Malta

**DOI:** 10.1038/s41431-020-00767-9

**Published:** 2021-01-07

**Authors:** Rebecca Borg, Maia Farrugia Wismayer, Karl Bonavia, Andrew Farrugia Wismayer, Malcolm Vella, Joke J. F. A. van Vugt, Brendan J. Kenna, Kevin P. Kenna, Neville Vassallo, Jan H. Veldink, Ruben J. Cauchi

**Affiliations:** 1 Centre for Molecular Medicine and Biobanking, Biomedical Sciences Building, University of Malta, Msida, Malta; 2 Department of Physiology and Biochemistry, Faculty of Medicine and Surgery, University of Malta, Msida, Malta; 3 Department of Neuroscience, Mater Dei Hospital, Msida, Malta; 4 Department of Neurology, Brain Center Rudolf Magnus, University Medical Center Utrecht, Utrecht, The Netherlands

**Keywords:** DNA sequencing, Motor neuron disease, Risk factors, Genetics of the nervous system, Genetic predisposition to disease

## Abstract

Genetic isolates are compelling tools for mapping genes of inherited disorders. The archipelago of Malta, a sovereign microstate in the south of Europe is home to a geographically and culturally isolated population. Here, we investigate the epidemiology and genetic profile of Maltese patients with amyotrophic lateral sclerosis (ALS), identified throughout a 2-year window. Cases were largely male (66.7%) with a predominant spinal onset of symptoms (70.8%). Disease onset occurred around mid-age (median age: 64 years, men; 59.5 years, female); 12.5% had familial ALS (fALS). Annual incidence rate was 2.48 (95% CI 1.59–3.68) per 100,000 person-years. Male-to-female incidence ratio was 1.93:1. Prevalence was 3.44 (95% CI 2.01–5.52) cases per 100,000 inhabitants on 31^st^ December 2018. Whole-genome sequencing allowed us to determine rare DNA variants that change the protein-coding sequence of ALS-associated genes. Interestingly, the Maltese ALS patient cohort was found to be negative for deleterious variants in *C9orf72*, *SOD1*, *TARDBP* or *FUS* genes, which are the most commonly mutated ALS genes globally. Nonetheless, ALS-associated repeat expansions were identified in *ATXN2* and *NIPA1*. Variants predicted to be damaging were also detected in *ALS2*, *DAO*, *DCTN1*, *ERBB4*, *SETX*, *SCFD1* and *SPG11*. A total of 40% of patients with sporadic ALS had a rare and deleterious variant or repeat expansion in an ALS-associated gene, whilst the genetic cause of two thirds of fALS cases could not be pinpointed to known ALS genes or risk loci. This warrants further studies to elucidate novel genes that cause ALS in this unique population isolate.

## Introduction

Amyotrophic lateral sclerosis (ALS) is an adult-onset, rapidly progressing, neurodegenerative disease. Onset is typically accompanied by clinical signs of upper and/or lower motor neuron degeneration and patients usually present with weakness in the bulbar muscles, only the limbs, or both regions simultaneously. About 15–20% of persons with ALS experience progressive cognitive decline, leading ultimately to dementia. The condition known as frontotemporal dementia results from the degeneration of the frontal and temporal lobes [1, 2]. The incidence of ALS in European populations is two to three cases per year per 100,000, whereas prevalence can reach 10/100,000 [3]. ALS is classified as familial (fALS) in the presence of a clear family history of the disease and sporadic (sALS) when this is absent. The rate of fALS among prospective population-based registries is about 5% [4]. Nonetheless, studies on twins and European case-control cohorts have found that heritability of ALS approximates 40% [5–7]. To date, variants in any of more than 40 genes have been reported to cause monogenic fALS with more than half of the cases explained by highly penetrant causal variants residing in *C9orf72* (23%), *SOD1* (19%), *TARDBP* (3%) or *FUS* (3%) [8]. Variants in these genes [8–10] and genetic risk loci that include *ATXN2* [11], *UNC13A* [12], *SARM1* [13], *C21orf2*, *SCFD1* and *MOBP* [13] have also been exposed in sALS cases. Genetic variants are estimated to contribute to between 14 and 17% of European sALS patients or those with a European ancestry [9, 10]. All this underscores the substantial contribution of genetic factors to ALS disease aetiology.

Genetic discoveries often lead to novel insights into the molecular mechanisms of ALS. For instance, clustering genes according to their known physiological functions identified ribostasis, proteostasis and cytoskeletal dynamics as key cellular pathways involved in ALS pathogenesis [14]. Importantly, discovery of genes is leading to the development of genotype-specific treatments [15]. Additional genetic factors remain to be found for ALS. The use of geographically and/or culturally isolated populations for mapping novel ALS genes is a strategy that remains relatively unexploited despite benefits that include founder effects, reduced genetic diversity and minimal environmental heterogeneity [16]. This spurred us to study the native population of Malta, a sovereign microstate in the middle of the Mediterranean Sea. Consisting of an archipelago of three inhabited islands (total area 316 km^2^), Malta’s population presently numbers about 514,564, based on Malta’s National Statistics Office (NSO) data in 2019. Population seeding events occurred more than 7000 years ago by settlers coming from neighbouring Sicily and based on mitochondrial DNA, Y-chromosome and autosomal DNA marker analyses, influences by colonisers in the centuries that followed were minimal [17–19]. The Maltese are the only European population that speak a Semitic language, further underscoring their relative isolation from other communities inhabiting Europe. Previous successes in gene mapping of rare diseases in the population of Malta are encouraging [20, 21]. Here, we first investigate the incidence and prevalence of ALS in the Maltese islands, in a 2-year period. Second, we perform an initial genetic survey by reporting on rare variants in protein-coding regions and consensus splice sites of all presently known monogenic ALS genes and genetic risk loci in Maltese ALS patients compared to matched controls.

## Materials and methods

### Participants

Participant surveillance and recruitment occurred throughout a 2-year window, from 2017 through 2018. Patients diagnosed with probable or definite ALS, referred by either the national Motor Neuron Disease association, consultant neurologists, general practitioners and neurophysiology units were invited to participate in our study. Alternatively, patients or their relatives made direct contact with our laboratory expressing willingness to participate in the study. Patient participants met the revised El Escorial criteria for ALS [22, 23]. Patients with fALS were identified as having a self-reported family history of ALS, or probable ALS, defined as the presence of at least one first-degree relative. In total, 24 patients were enrolled in this study. Blood sampling was excluded for one sALS case in view of the patient’s deteriorating condition. Affected family members for all fALS cases were deceased precluding us from sampling them. Controls, which were ascertained in a roughly 2:1 case-control ratio, matched patients for age, sex and geographical region. Ethical approval for the collection of samples, study design and the creation of the Malta ALS/MND Register and Biobank was given by the Research Ethics Committee of the University of Malta. Written informed consent to participate was sought from all patients and/or family members as well as controls.

### Phenotypic information

Phenotypic information was gathered via a detailed questionnaire in addition to a clinical examination. Each sample is therefore accompanied by a core dataset that includes age, sex, occupation, site of onset, date of disease onset, family history, ALS Functional Rating Scale-revised (ALSFRS-R) score, muscle tone for both upper and lower limbs, muscle power graded according to the MRC scale, and status of reflexes. Information on possible environmental risk factors including physical activity, cigarette smoking and alcohol consumption was also gathered. A biochemical assay to test creatine kinase (CK) levels at recruitment was also utilised.

### Incidence and prevalence calculations

The denominator for the calculation of the incident rate was the sum of total population of Malta in 2017 and 2018. During the study period, the population of Malta increased from 475,701 to 493,559. Separate incident rates for males and females as well as specific age groups were also calculated. Population numbers were derived from NSO data. The prevalence rate was estimated on 31^st^ December 2018. Confidence limits for incidence were calculated assuming a Poisson distribution.

### Whole-genome sequencing

Extraction of DNA occurred from whole EDTA-containing venous blood samples using the QIAamp DNA Mini QIAcube Kit and DNA integrity was measured using the Quantus fluorometer. DNA was whole-genome sequenced by the BGISEQ-500 platform (BGI, Hong Kong, China) to generate 100 bp paired-end reads with an average depth of 30×. Reads were aligned to the GRCh37 (HG19) reference genome using Burrows–Wheeler Aligner software. Single nucleotide variant (SNV) and small insertion and deletion (indel) calling and quality filtering were performed using the Genome Analysis Toolkit (GATK). The ExpansionHunter tool was used to analyse repeat sizes of *ATXN2* (NM_002973.3:c.496_498CAG), *C9orf72* (NM_001256054.2:c.-45+163GGGGCC) and *NIPA1* (NM_144599.4:c.24_26GGC) [24]. In order to estimate the genetic ancestry in relation to reference maps of diverse populations, principal-component analysis (PCA) was performed on LASER with results plotted using the LASER Server plot facility (https://laser.sph.umich.edu/). HomozygosityMapper (www.homozygositymapper.org) [25] was used to map runs of homozygosity (ROHs).

### Variant analysis

We searched for and analysed protein-coding and splice-site altering variants and indels in 58 established ALS causative or risk genes (Table 1). We restricted analyses to variants with European minor allele frequency (MAF) ≤0.01, which corresponds roughly to the European frequency of the recently discovered ALS risk NM_004984.2:c.2957C>T; p.(Pro986Leu) allele in the *KIF5A* gene [26]. Where available, variants were then annotated with information from the dbSNP database including European-specific MAF estimates from the Genome Aggregation Database (gnomAD). Allele frequencies for ALS cases and controls within the Project MinE dataset were extracted from the Project MinE databrowser [27]. To determine variant pathogenicity, MetaSVM and MetaLR, two ensemble-based prediction methods integrating multiple scoring systems, were used in view of their superior predictive ability relative to other methods [28]. Variants were considered as damaging if outcomes of both methods concurred. Indels and splice-site acceptor/donor variants were automatically classified as deleterious. Variants and the associated phenotypes have been submitted to the ClinVar database (https://www.ncbi.nlm.nih.gov/clinvar/) with accession numbers SCV001426191, SCV001426206-SCV001426210, SCV001426221-SCV001426223 and SCV001437161-SCV001437192. New variants detected in the Maltese case-control cohort were submitted to the dbSNP database (https://www.ncbi.nlm.nih.gov/snp/) with the submission SNP IDs of ss2137544106, ss3986090479, ss3986090480, and ss3986090481.

**Table 1:** ALS-associated genes investigated in this study, and their basic characteristics.

Gene	Chromosome locus	Genetic effect	Transcript length (kbp)	Putative pathway/function
*ALS2*	2q33.1	AR	6.68	Vesicular trafficking
*ANG*	14q11.2	AD	1.22	Angiogenesis
*ANXA11*	10q22.3	AD	6.97	Proteostasis
*ATXN2*	12q24.12	AD, RF	4.7	Ribostasis; endocytosis
*C21orf2/CFAP410*	21q22.3	AD, RF	2.21	Cytoskeletal organisation; cilia formation
*C9orf72*	9p21.2	AD	3.34	Autophagy; intracellular trafficking; proteostasis; nucleocytoplasmic transport
*CAPN14*	2p23.1	RF	3.79	Apoptosis; cytoskeletal dynamics
*CCNF*	16p13.3	AD	4.23	Proteostasis
*CHCHD10*	22q11.23	AD	1.17	Autophagy; neuroinflammation
*CHMP2B*	3p11.2	AD	2.59	Vesicular trafficking; proteostasis
*CYLD*	16q12.1	AD	8.5	Deubiquitinase activity
*DAO*	12q24.11	AD	1.69	D-serine regulation
*DCTN1*	2p13.1	AD	4.5	Vesicular trafficking; proteostasis
*DDX20*	1p13.2	RF	3.6	Ribostasis
*DNAJC7*	17q21.2	AD	1.81	Proteostasis
*ELP3*	8p21.1	RF	3.15	Ribostasis; cytoskeletal integrity
*ERBB4*	2q34	AD	12.1	Cell signalling
*EWSR1*	22q12.2	AD	2.65	Ribostasis
*FIG4*	6q21	AD	3.01	Vesicular trafficking
*FUS*	16p11.2	AD	5.12	Ribostasis
*GGNBP2*	17q12	RF	2.82	Cell signalling
*GLE1*	9q34.11	AR	3.32	Ribostasis
*GLT8D1*	3p21.1	AD	1.85	Proteostasis
*GPX3*	5q33.1	RF	1.6	Oxidative stress
*GRN*	17q21.31	AD	2.13	Cell growth
*HNRNPA1*	12q13.13	AD	1.84	Ribostasis
*HNRNPA2B1*	7p15.2	AD	3.66	Ribostasis
*KIF5A*	12q13.3	AD	5.78	Vesicular trafficking
*MAPT*	17q21.31	AD	6.82	Cytoskeletal organisation
*MATR3*	5q31.2	AD	4.84	Ribostasis
*MOBP*	3p22.1	RF	1.34	Structural constituent of myelin sheath
*NEFH*	22q12.2	AD	3.78	Axonal transport
*NEK1*	4q33	AD	5.65	DNA damage repair
*NIPA1*	15q11.2	RF	6.55	Vesicular trafficking
*OPTN*	10p13	AD, AR	3.52	NfkB signal transduction; autophagy
*PFN1*	17p13.2	AD	1.29	Cytoskeletal organisation; axonal growth and transport
*PRPH*	12q13.12	AD	1.8	Cytoskeletal organisation
*SARM1*	17q11.2	RF	10.27	Cell signalling
*SCFD1*	14q12	RF	2.36	Vesicular trafficking
*SETX*	9q34.13	AD	11.1	Ribostasis
*SIGMAR1*	9p13.3	AR	1.67	Signal transduction amplification
*SMN1*	5q13.2	RF	1.55	Ribostasis
*SMN2*	5q13.2	RF	1.55	Ribostasis
*SOD1*	21q22.11	AR, AD	0.97	Oxidative stress; proteostasis
*SPAST*	2p22.3	AD	5.28	Endocytosis
*SPG11*	15q14	AR	7.77	Cytoskeletal organisation; vesicular transport
*SQSTM1*	5q35.3	AD	2.84	Autophagy; neuroinflammation
*SS18L1*	20q13.33	AD	4.55	Ribostasis
*TAF15*	17q12	AR, AD	2.16	Ribostasis
*TARDBP*	1p36.22	AD	5.37	Ribostasis
*TBK1*	12q14.2	AD	3.02	Autophagy, inflammation
*TIA1*	2p13.3	AD	4.63	Ribostasis
*TNIP1*	5q33.1	RF	2.79	NfκB signal transduction
*TUBA4A*	2q35	AD	2.05	Cytoskeletal organisation; axonal transport
*UBQLN2*	Xp11.21	XD	4.23	Proteostasis
*UNC13A*	19p13.11	RF	9.84	Neurotransmitter release regulation
*VAPB*	20q13.32	AD	7.94	Proteostasis
*VCP*	9p13.3	AD	3.75	Proteostasis

### Statistics

Comparisons between means were made with the unpaired, two-tailed Student *t* test, whereas comparison between categorical variables was made with *χ*^2^ test. A *p* value <0.05 was considered significant. Data were processed with GraphPad Prism v8.4.0 software.

## Results

### Baseline characteristics

The key characteristics of patients and controls are detailed in Table 2. ALS cases were largely male with a predominant spinal onset of symptoms. Bulbar onset ALS had a slightly higher occurrence in females (57.2%). Disease onset occurred around mid-age with median age at onset being lower in females (59.5 years) compared to males (64 years). Only one male and female patient was under 45 years old (8.3%). Early ALS (≤55 years) was more frequently spinal at onset (spinal/bulbar ratio = 9:1). A family history of ALS was recorded for a minority of cases (Table 2). However, considering their familial aggregation with ALS [29–31], the inclusion of both a family history of dementia and neuropsychiatric endophenotypes-like schizophrenia or psychosis, increases the proportion of fALS to 37.5% (9/24 patients). The mean duration of the illness was 44.5 ± 28.3 SD months, with males having a faster progression compared to females (29.6 ± 8.8 SD months vs. 66.8 ± 33.4 SD months, *p* = 0.0065). Site of disease onset did not influence disease duration (spinal onset = 43.5 ± 32.4 SD months, bulbar onset = 43.7 ± 19.8 SD months, *p* = NS).

**Table 2:** Baseline characteristics of ALS patients and controls.

	ALS (*n* = 24)	Controls (*n* = 13)
Sex		
Male, % (*n*)	66.7 (16)	46.2 (6)
Female, % (*n*)	33.3 (8)	53.8 (7)
Type		
Familial, % (*n*)	12.5 (3)	–
Sporadic, % (*n*)	87.5 (21)	–
Age at onset		
Mean year ± SD (range)	59.3 ± 13.2 (27–80)	–
Age at recruitment		
Mean year ± SD (range)	63.5 ± 11.6 (30–82)	71.2 ± 10.9 (52–90)
ALSFRS-R score, mean ± SD (range)	25.6 ± 11.1 (11.5–44)	47.9 ± 0.3 (47–48)
Site of onset		
Spinal, % (*n*)	70.8 (17)	–
Bulbar, % (*n*)	25 (6)	–
Both, % (*n*)	4.2 (1)	
Survival, mean years ± SD (range)	5.5 ± 5.1 (2-20)	–
Deceased, % (*n*)	62.5 (15)	–
Cognitive status		
Normal, % (*n*)	95.8 (23)	100 (13)
Impaired, % (*n*)	4.2 (1)	0 (0)
Environmental risk factors		
Heavy smoking, % (*n*)	33.3 (8)	23.1 (3)
Strenuous activity, % (*n*)	54.2 (13)	23.1 (3)
Excessive alcohol consumption, % (*n*)	8.3 (2)	7.8 (1)
CK levels at recruitment, IU/L^a^		
Mean in males ± SD (range)	311.2 ± 259.7 (15–863)	–
Mean in females ± SD (range)	180.8 ± 117.5 (38–336)	–

*ALSFRS-R* Amyotrophic Lateral Sclerosis Functional Rating Scale-Revised, *CK* creatine kinase.

^a^Normal CK range = 39–308 U/L (males), 26–192 U/L (females).

**Fig. 1:**
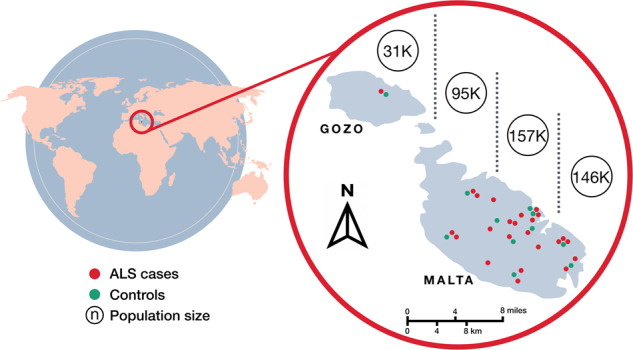
Geographical distribution of ALS cases and controls for individuals born on the Maltese islands. Population size of the four regional divisions (separated by dotted lines) is based on NSO data in 2017.

One-third of the ALS patients recruited had a history of heavy smoking and more than half reported an occupation associated with strenuous activity, both of which have been implicated as environmental risk factors for ALS [32, 33]. Alcohol abuse in the patient cohort was minimal. In view that a number of years have elapsed from onset up to recruitment, the levels of CK in the patient cohort were on average only slightly above the normal range. The mean ALSFRS-R score in ALS recruits was nearly half that recorded for the control subjects with the score in the latter nearing 48, the maximum expected in healthy individuals. The distribution of cases and controls throughout the Maltese islands are displayed in Fig. 1. A higher population density in the southeast of mainland Malta, most probably explains the increase in the number of cases in this geographic region relative to other regions.

### Incidence and prevalence rates

**Fig. 2:**
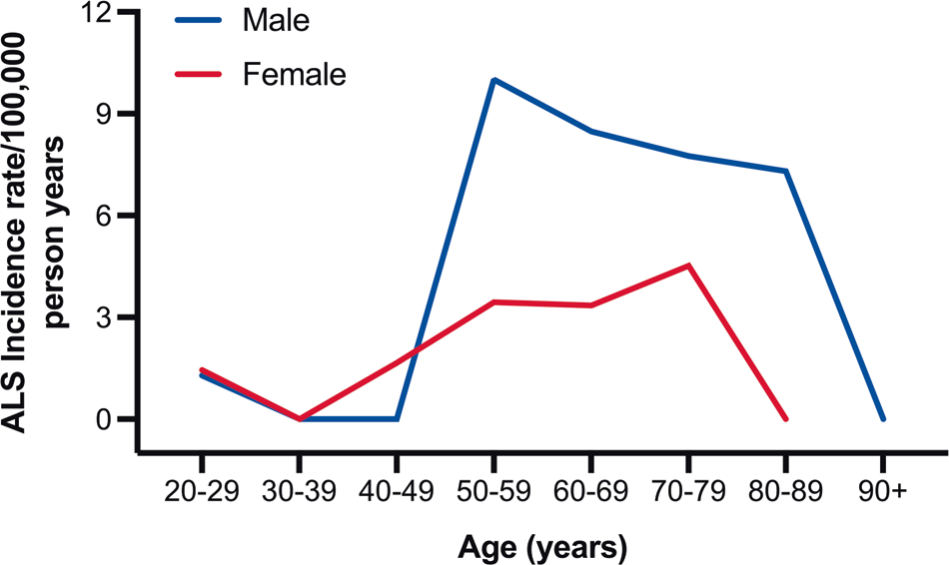
Change in incidence rate of ALS with age in Malta. Incidence rate increases with age and was higher for males compared to females across all age groups after 49 years. Peaks occur in the 50–59 age group for men and in the 70–79 age group for women.

The annual incidence rate for ALS in the 2017–2018 period was 2.48/100,000 person-years (95% CI 1.59–3.68). The male-to-female incidence ratio was 1.93:1. Hence, incidence rate was higher for men (3.25, 95% CI 1.86–5.28) than for women (1.68, 95% CI 0.72–3.31), and this trend occurred across all age groups after 49 years (Fig. 2). For both men and women, the incidence increased with age but declined after age 79. Peaks occurred in the 50 to 59 age group among men and in the 70 to 79 age group among women. A total of 17 patients (male = 12, female = 5) were alive at the prevalence date (31^st^ December 2018), corresponding to a crude prevalence of 3.44/100,000 (95% CI 2.01–5.52). Similarly, the prevalence in males (5.16, 95% CI 2.74–8.83) was higher than that in females (1.66, 95% CI 0.45–4.24), leading to a male-to-female prevalence ratio of 3.11:1. There was no difference in the mean age of onset and site of onset between prevalent and incident patients.

### Genetic ancestry

**Fig. 3:**
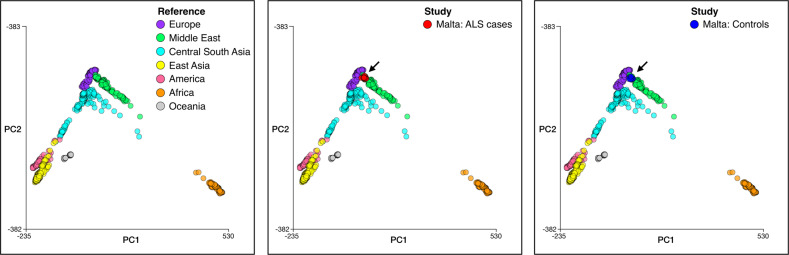
Ancestry of Maltese ALS cases and controls compared to the HGDP reference panel. *Left panel*, Reference PCA coordinates for samples from the HGDP reference panel. *Middle panel*, Maltese ALS cases mapped to the reference PCA coordinates. *Right panel*, Maltese controls mapped to the reference PCA coordinates.

PCA analysis of the Maltese case-control cohort showed that cases and controls were within 3 standard deviations of the mean for the combined samples along principal components (PC) 1–4, hence ensuring adequate genetic matching. Mapping the Maltese ALS patient and control samples to the reference PCA coordinates for samples from the Human Genome Diversity Panel (HGDP) shows overlap with the portion of the Middle-Eastern cluster that neighbours the European cluster (Fig. 3). A genetic affinity with Middle East populations is also apparent in Sicilians [34, 35], further supporting a common genetic ancestry for populations inhabiting the Mediterranean islands of Malta and Sicily.

### Repeat expansions in *C9orf72*, *ATXN2* and *NIPA1* genes

**Fig. 4:**
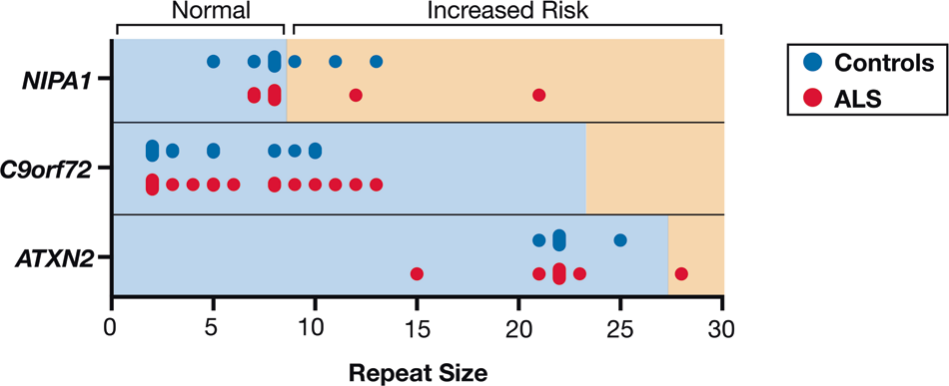
Expansion repeat size in ALS cases and controls for *NIPA1*, *C9orf72* and *ATXN2* genes. Scatter plot showing distribution and frequency of repeat sizes, indicated by circles.

Despite studies which show that *C9orf72* is the major gene that is mutated in the European ALS population [8, 36, 37], we did not identify pathogenic hexanucleotide (GGGGCC) repeat expansions (≥24) in *C9orf72* in either fALS or sALS cases (Fig. 4). The expansion repeat size ranged from 2 to 10 in controls and from 2 to 13 in ALS patients. Consistent with previous studies [36, 37], a repeat length of 2 was the most predominant in either group (42.3% in controls and 52.2% in ALS cases). In addition to *C9orf72*, repeat expansions in other genes including *ATXN2* and *NIPA1* have been associated with increased risk of ALS [11, 38]. We identified one male patient with fALS that possessed *ATXN2* ALS-associated trinucleotide repeat expansions (28 repeats in length) in the homozygous state (Fig. 4). At the age of 67 years, this patient first experienced bilateral leg weakness that progressed. He subsequently developed dysarthria and succumbed to the disease within 2 years of disease onset. This patient was also the only one in our cohort that showed signs of cognitive impairment. Family history was notable for a deceased sister who had ALS with early onset in her late 30s, and a deceased mother who had dementia (DNA samples were not available for study). The pedigree is shown in Supplementary Fig. S1. The maximum *ATXN2* repeat size observed in healthy controls was 25, encountered in the heterozygous state, in one subject. All remaining ALS patients and controls had repeat lengths ≤23. As was reported previously [11, 39], a repeat length of 22, detected mostly in the homozygous state, was the most abundant *ATXN2* allele (88.5% in controls and 84.8% in ALS patients). Considering *NIPA1*, we found one male patient with sALS who had an expanded [21] GCG repeat motif in the heterozygous state. The patient had a late age of onset (73 years), experiencing bilateral weakness first in the lower limbs and then in the upper limbs. Survival was shorter (<2.5 years) compared with the median survival (3.5 years) in our ALS cohort. In controls and in the remaining ALS cases, repeat length was variable ranging from 5 to 13. Similar to previous findings [38, 40], the most frequent alleles consisted of either 7 or 8 repeats, with respective allele frequencies being 26.1% and 67.4% in ALS patients, and 7.7% and 73.1% in controls.

### Genetic variants in known ALS-associated genes

Interestingly, the Maltese ALS patient cohort was found to be negative for non-synonymous or splice-site altering SNVs in the *SOD1*, *TARDBP* or *FUS* genes, which are the most commonly mutated ALS genes, in that order, following *C9orf72* [8]. After examining 58 ALS-associated genes in our patient and control cohort, we identified 35 rare (European MAF ≤ 0.01) coding variants that were present in Maltese ALS patients and absent in controls (Table 3). Three SNVs in *DDX20*, *EWSR1* or *GLE1* were not found in the dbSNP (v141) database. The NM_001003722.1:c.2078C>T; p.(Ser693Phe) variant in *GLE1* was however reported in a recent study as a founder mutation in Maltese that on homozygosis induces a motor dysfunction syndrome that presents at childhood [21]. Variants predicted to be damaging by both MetaSVM and MetaLR were detected in *ALS2*, *DAO*, *DCTN1*, *ERBB4*, *SCFD1* and *SPG11* (Table 3). All were detected in patients with sALS. The NM_001917.4:c.250G>A; p.(Ala84Thr) variant in *DAO* was detected in two patients, whereas one female patient possessed deleterious variants in more than one gene (*DAO* and *DCTN1*).

**Table 3:** Rare non-synonymous single-nucleotide variants and indels found in Maltese ALS patients.

				Prediction			Project MinE Allele Frequency				
Gene	cDNA change	Protein change	dbSNP^141^ ID	MetaSVM	MetaLR	European gnomAD MAF	ALS cases	Controls	No. of patients	ALS type	Zygosity
*ALS2*	NM_020919.3:c.3206G>A	p.(Gly1069Glu)	rs200706696	Damaging	Damaging	0.0003	0.000376	0	1	sALS	het
*ATXN2*	NM_002973.3:c.2950A>C	p.(Ile984Leu)	rs1338140819	Tolerated	Tolerated	0.00001	NA	NA	1	sALS	het
*C21orf2*	NM_001271441.1:c.661G>A	p.(Ala221Thr)	rs746114248	Tolerated	Tolerated	0.0000	NA	NA	1	sALS	het
*CAPN14*	NM_001145122.1:c.1249C>T	p.(Leu417Phe)	rs181906086	Tolerated	Tolerated	0.0183	0.004810	0.005259	1	fALS	het
*DAO*	NM_001917.4:c.250G>A	p.(Ala84Thr)	rs781658657	Damaging	Damaging	0.00001	NA	NA	2	sALS	het
*DCTN1*	NM_004082.4:c.1484G>A	p.(Arg495Gln)	rs17721059	Tolerated	Tolerated	0.014	0.018945	0.019013	1	fALS	het
*DCTN1*	NM_004082.4:c.586A>G	p.(Ile196Val)	rs55862001	Tolerated	Tolerated	0.0055	0.005720	0.006885	1	sALS	hom
*DCTN1*	NM_004082.4:c.1864A>T	p.(Ile622Phe)	rs1328116832	Damaging	Damaging	0.00001^a^	NA	NA	1	sALS	het
*DDX20*	NM_007204.4:c.2237T>C	p.(Leu746Ser)	NA	Tolerated	Tolerated	NA	NA	NA	1	fALS	het
*DNAJC7*	NM_001144766.2:c.2T>C	p.(Met1?)	rs371236469	Tolerated	Tolerated	0.00005	0.000075	0	1	sALS	het
*ERBB4*	NM_005235.2:c.3814G>A	p.(Gly1272Arg)	rs371332509	Damaging	Damaging	0.00001	NA	NA	1	sALS	het
*ERBB4*	NM_005235.2:c.1122T>G	p.(His374Gln)	rs76603692	Tolerated	Tolerated	0.0009	0.001955	0.002429	1	sALS	het
*ERBB4*	NM_005235.2:c.3176T>C	p.(Met1059Thr)	rs373685875	Tolerated	Tolerated	0.00001	NA	NA	1	sALS	het
*EWSR1*	NM_013986.3:c.1798G>A	p.(Asp600Asn)	NA	Tolerated	Tolerated	NA	NA	NA	1	fALS	het
*EWSR1*	NM_013986.3:c.1408G>A	p.(Gly470Ser)	rs41311143	Tolerated	Tolerated	0.0121	0.013158	0.013754	1	sALS	het
*GLE1*	NM_001003722.1:c.2078C>T	p.(Ser693Phe)	NA	Tolerated	Tolerated	NA	NA	NA	1	sALS	het
*KIF5A*	NM_004984.2:c.2957C>T	p.(Pro986Leu)	rs113247976	Tolerated	Tolerated	0.018	0.020069	0.014777	1	sALS	het
*MOBP*	NM_001278322.1:c.586C>A	p.(Arg196Ser)	rs1188260744	Tolerated	Tolerated	0.0000	NA	NA	1	sALS	het
*NEFH*	NM_021076.3:c.2009T>A	p.(Val670Glu)	rs190692435	Tolerated	Tolerated	0.00676	NA	NA	2	sALS, fALS	het
*NEK1*	NM_001199397.1:c.107A>G	p.(Asn36Ser)	rs1404362599	Tolerated	Tolerated	NA	NA	NA	1	sALS	het
*SARM1*	NM_015077.4:c.1501T>C	p.(Tyr501His)	rs144613221	Damaging	Tolerated	0.00228	0.003458	0.002832	1	sALS	het
*SCFD1*	NM_016106.3:c.209T>C	p.(Ile70Thr)	rs61754480	Damaging	Damaging	0.00403	0.004660	0.003843	1	sALS	het
*SCFD1*	NM_016106.3:c.1297A>G	p.(Thr433Ala)	rs61754285	Tolerated	Tolerated	0.01788	0.018641	0.023058	2	sALS	het
*SETX*	NM_015046.5:c.7640T>C	p.(Ile2547Thr)	rs151117904	Tolerated	Tolerated	0.0032	0.007066	0.004652	1	sALS	het
*SETX*	NM_015046.5:c.2425A>G	p.(Ile809Val)	rs906452681	Tolerated	Tolerated	0.00003	0	0.000202	1	sALS	het
*SETX*	NM_015046.5:c.5308_5311del	p.(Glu1770Ilefs*15)	rs750959420	Damaging^b^	Damaging^b^	0.0000	0.000075	0	1	sALS	het
*SPG11*	NM_025137.3:c.1618C>T	p.(Arg540Cys)	rs758046989	Damaging	Damaging	0.00001	NA	NA	1	sALS	het
*SPG11*	NM_025137.3:c.6759C>G	p.(Asp2253Glu)	rs141818132	Tolerated	Tolerated	0.00003	0	0	1	sALS	het
*SPG11*	NM_025137.3:c.1698T>G	p.(Asp566Glu)	rs79708848	Tolerated	Tolerated	0.01798	0.016165	0.014563	1	sALS	het
*SPG11*	NM_025137.3:c.16G>A	p.(Gly6Arg)	rs200573434	Tolerated	Tolerated	0.00208	0.002330	0.001619	1	sALS	het
*SPG11*	NM_025137.3:c.3037A>G	p.(Lys1013Glu)	rs111347025	Tolerated	Tolerated	0.01224	0.016912	0.017800	1	sALS	het
*SPG11*	NM_025137.3:c.3425C>G	p.(Ser1142Cys)	rs201082396	Tolerated	Tolerated	0.0001	NA	NA	1	fALS	het
*SPG11*	NM_025137.3:c.2656T>C	p.(Tyr886His)	rs139687202	Tolerated	Tolerated	0.0001	0.000225	0.000405	1	sALS	het
*SPG11*	NM_025137.3:c.7256A>G	p.(Lys2419Arg)	rs76116949	Tolerated	Tolerated	0.0001	0.000075	0	1	sALS	het
*TNIP1*	NM_001252390.1:c.437C>T	p.(Ala146Val)	rs2233289	Tolerated	Tolerated	0.0103	0.012103	0.013754	1	sALS	het

*dbSNP* Single-Nucleotide Polymorphism database, *gnomAD* Genome Aggregation Database, *MAF* minor allele frequency, *NA* not available, *het* heterozygote.

^a^Data from Trans-Omics for Precision Medicine (TopMed) Program.

^b^Indel was automatically considered deleterious.

Analysis of indels allowed us to identify a rare deletion in *SETX*, detected in the heterozygous state in a sALS case (Table 3). This patient presented with upper limb weakness at the age of 70. One year later, on follow-up, weakness spread to the lower limbs. The deletion is predicted to result in a frameshift, consequently producing a truncated *SETX* protein lacking the helicase domain. It is noteworthy that the damaging *ALS2*, *SCFD1,* and *SETX* variants detected in Maltese sALS patients were found to have higher allele frequencies in ALS patients within the Project MinE case-control dataset [27], thereby underscoring their probable pathogenicity (Table 3). Rare SNVs or indels that were unique to controls or that were shared by ALS patients and controls are listed in Supplementary Table S1. One SNVs in *FIG4* was not found in the dbSNP (v141) database. No homozygous stretches were overrepresented in sALS patients compared to controls (Supplementary Fig. S2). To estimate the genetic risk for ALS in the Maltese population, we determined that the percentage of sALS cases caused by rare and potentially deleterious variants (absent in controls) in at least one ALS-associated gene was 40% (8/20 patients). Two fALS cases did not carry any mutations in known ALS genes or risk loci, hence, warranting further studies to elucidate novel genes that cause ALS.

## Discussion

In our work, we investigated the characteristics of Maltese ALS patients, described their genetic profile, and determined the incidence and prevalence of ALS on the Maltese islands. It is interesting that the population-specific aspects of our ALS cases overlap those reported for other neighbouring European populations, especially those in the Mediterranean including the island of Sicily [41] and the southern region of Puglia [42, 43] in Italy, Tunisia [44] and Cyprus [45]. Although the male preponderance in ALS is virtually universal [46, 47], it is noteworthy that in these specific populations as well as in Malta, age at disease onset is higher in males than in females. This is in contrast to northern populations of the Mediterranean basin including Catalonia in Spain [48], and the regions of Emilia Romagna [49], Liguria [50] or Friuli-Venezia-Giulia [51] in Italy. Incidence and prevalence of ALS in Malta is similar to the European median [3].

We report a higher percentage (12.5%) of fALS cases in Malta, close to that reported for the northern Italian population of Liguria (10%) [50] but nearly half that reported for the island of Sardinia (26.7%) [52]. Nonetheless, similar to previous studies [53], relaxing the stringent criteria by including neurological conditions in kindreds that have a genetic overlap with ALS [54], can increase the fALS percentage in Malta by threefold (37.5%). In agreement, considering the Maltese sALS subset, we showed that, compared to other European populations [10] or populations of European ancestry [9], a higher proportion of seemly sALS patients are probably genetically determined.

Intriguingly, rare deleterious variants in the major ALS genes, including *C9orf72*, *SOD1*, *TARDBP* and *FUS*, were absent in Maltese ALS patients. This finding confirms the presence of a North–South gradient in the frequency of mutations within these genes across Europe. Hence, *C9orf72* or *SOD1* mutations in fALS are highest in northern European countries like Belgium and Finland, whereas a relatively low frequency is recorded in the south of Europe including Spain and mainland Italy [8]. A similar situation can be observed for *TARDBP* and *FUS* [8]. Our study thus underscores the marked differences that exists between ethnic groups and geographical regions with respect to the genes that are commonly implicated in ALS.

Maltese ALS patients nevertheless possessed deleterious alleles in ‘minor’ ALS genes including *ALS2*, *ATXN2*, *DAO*, *DCTN1*, *ERBB4*, *NIPA1*, *SETX*, *SCFD1* and *SPG11*. *ALS2* and *SPG11* have been associated with juvenile-onset ALS only under a recessive disease model [55–58]. In this context, since we observed variants in the *ALS2* and *SPG11* genes solely in heterozygous configurations and in patients which had adult-onset ALS, it is likely that these alleles were not disease causing in the patients that possessed them. However, considering that ALS has an oligogenic basis [59], a modifying or additive effect cannot be excluded. The same can be said for *SCFD1*, which has only been recently identified as a risk locus [13], and for which we report a damaging variant in an ALS patient with a young age of onset (27 years) and whose disease progression is exceptionally slow.

*ATXN2*, *DAO*, *DCTN1*, *ERBB4*, *NIPA1* and *SETX* have all been previously associated with ALS having an autosomal dominant mode of inheritance [11, 38, 40, 60–63]. Damaging alleles discovered in our patient cohort, which specifically target these genes, are most probably causative. It is interesting to note that the *ERBB4* c.3814G>A; p.(Gly1272Arg) variant reported in this study is extremely close to the one reported in a Japanese sALS individual [c.3823C>T; p.(Arg1275Trp)], both of which are located in the C-terminal domain of the protein, close to multiple phosphorylation sites, which mediate downstream signalling pathways [61]. Although *SETX* variants have been initially discovered in juvenile-onset ALS patients [62], reports have since described damaging alleles in patients with adult-onset ALS [10, 64]. This is in line with our study, hence, the Maltese ALS patient possessing a *SETX* deletion had a late age of onset similar to the one reported in a previous case study [64].

Our findings have important implications. Incidence and prevalence of ALS in Malta as well as patient population aspects overlap those of neighbouring countries. However, supported by genetic ancestry results, the genetic architecture of ALS in Malta appears to be different from the European average underscoring genetic isolation imposed by geography. This combined with the lack of an identified genetic factor in two-thirds of Maltese fALS cases, encourages further studies aimed at discovering novel ALS genes. Our ‘preliminary’ data excludes the possibility that these patients have deleterious variants in a set of genes associated with other motor neuron disorders including hereditary ataxias, and hereditary motor and sensory neuropathies (data not shown). Finally, variants described in this work should spur the generation of animal models to confirm causation and better understand disease mechanisms [65]. This is imperative especially for ‘minor’ ALS genes, given that they are relatively less studied than ‘major’ genes, but which are nonetheless consequential in specific populations.

## Supplementary information

Supplementary Material
