# Supplementary material for: Genetic analysis of ALS cases in the isolated island population of Malta
Source: Eur J Hum Genet. 2021 Jan 7;29(4):604–14. doi: 10.1038/s41431-020-00767-9 (PMC8115635; doi:10.1038/s41431-020-00767-9)
Supplement: Supplementary file 1 — Supplementary Material [file 41431_2020_767_MOESM1_ESM.pdf]

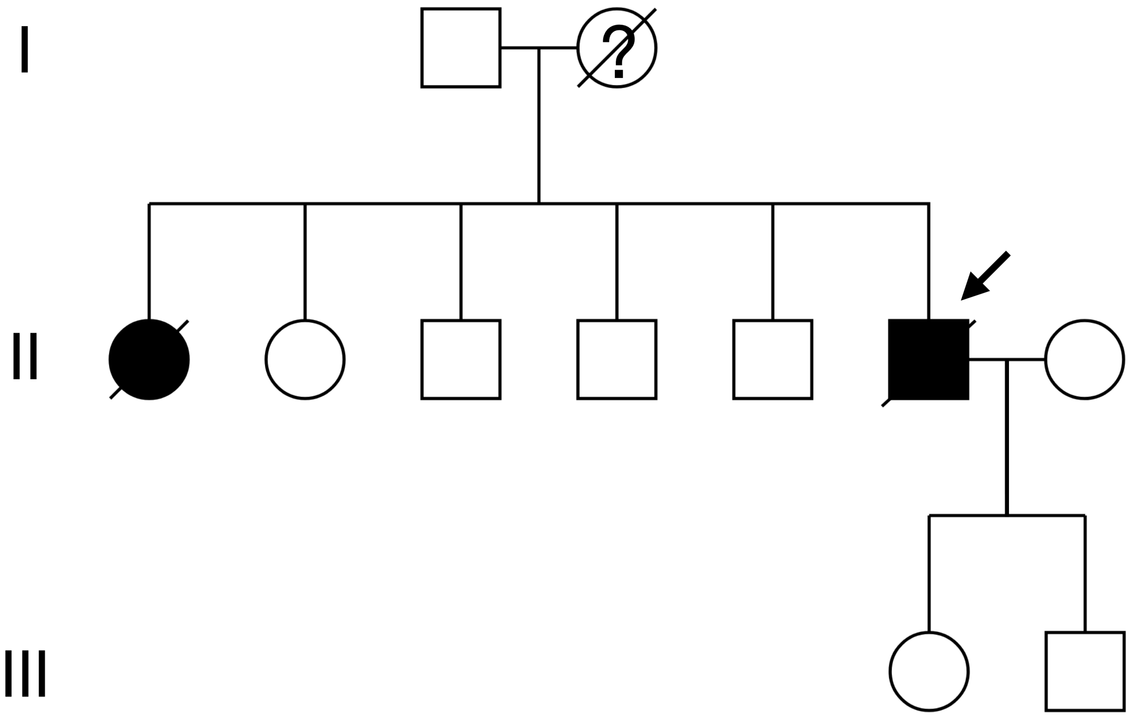

Figure S1. Pedigree chart of a fALS case with ALS-associated expanded *ATXN2* repeats. Filled symbols indicate affected individuals. The arrow indicates the proband.

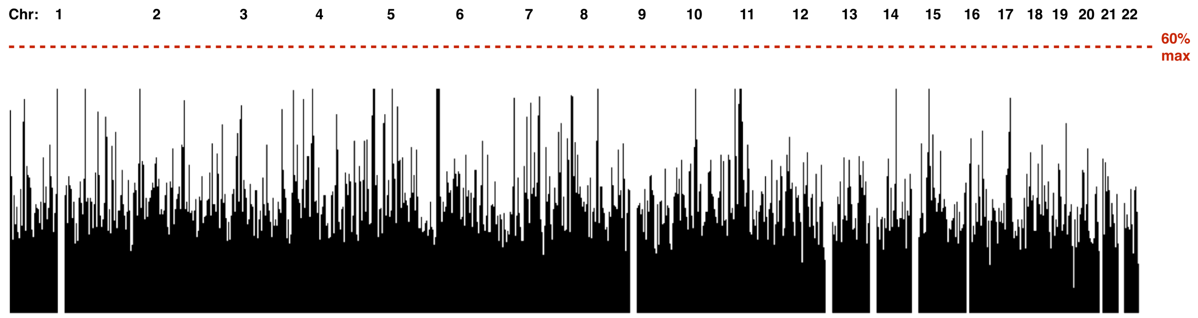

Figure S2. Homozygosity mapping of Maltese sALS patients. Mapping with allowed genetic heterogeneity did not reveal any genomic intervals that satisfied accepted criteria for a significant excess of homozygosity (HomozygosityMapper score >80% of max value). Chromosome numbers are indicated on top.

Gene	cDNA change	Protein change	dbSNP[141] ID	Prediction		European gnomAD MAF	Project MinE Allele Frequency		No. of patients	ALS type	No. of controls
				MetaSVM	MetaLR		ALS cases	Controls			
ATXN2	NM_002973.3:c.743G>A	p.(Ser248Asn)	rs7969300	Tolerated	Tolerated	0.0031	0.006239	0.005057	0	NA	2
ATXN2	NM_002973.3:c.1507G>C	p.(Val503Leu)	rs771843383	Tolerated	Tolerated	0.00002	NA	NA	0	NA	1
CCNF	NM_001761.2:c.2122G>C	p.(Ala708Pro)	rs754766175	Tolerated	Tolerated	NA	NA	NA	0	NA	1
CCNF	NM_001761.2:c.1188G>T	p.(Glu396Asp)	rs36008785	Tolerated	Tolerated	0.0001	0.000827	0.000202	0	NA	1
CYLD	NM_015247.2:c.665C>A	p.(Thr222Lys)	rs587778225	Tolerated	Tolerated	0.00011	NA	NA	0	NA	2
ERBB4	NM_005235.2:c.3859G>A	p.(Glu1287Lys)	rs367568427	Damaging	Damaging	0.00003	NA	NA	0	NA	1
FIG4	NM_014845.5:c.434C>T	p.(Pro145Leu)	NA	Tolerated	Tolerated	NA	NA	NA	0	NA	1
GLE1	NM_001003722.1:c.5C>G	p.(Pro2Arg)	rs150246404	Tolerated	Tolerated	0.0008	0.000752	0.000607	0	NA	1
KIF5A	NM_004984.2:c.2927C>T	p.(Thr976Ile)	rs139801016	Tolerated	Tolerated	0.0001	0.000301	0.000202	1	fALS	1
NEFH	NM_021076.3:c.1684C>G	p.(Pro562Ala)	rs530872313	Tolerated	Tolerated	0.00001	0.000230	0.000205	0	NA	1
NEK1	NM_001199397.1:c.1535C>T	p.(Ala512Val)	rs771824152	Tolerated	Tolerated	0.00006	NA	NA	0	NA	1
SETX	NM_015046.5:c.3047T>C	p.(Ile1016Thr)	rs200856903	Damaging	Damaging	0.00001	NA	NA	1	sALS	1
SPG11	NM_025137.3:c.820G>A	p.(Val274Ile)	rs543316224	Tolerated	Tolerated	0.00001	NA	NA	0	NA	1
SS18L1	NM_198935.2:c.961G>A	p.(Ala321Thr)	rs36106901	Tolerated	Tolerated	0.00042	0.001353	0.000607	2	sALS	2
TIA1	NM_022173.2:c.1070A>G	p.(Asn357Ser)	rs116621885	Tolerated	Tolerated	0.0125	0.009847	0.005461	0	NA	1
TNIP1	NM_001252390.1:c.1748C>T	p.(Pro583Leu)	rs888695021	Tolerated	Tolerated	0.00001[a]	NA	NA	0	NA	1
TNIP1	NM_001252390.1:c.1049C>T	p.(Thr350Ile)	rs369725837	Tolerated	Tolerated	0.00002	0	0.000405	0	NA	1
UNC13A	NM_001080421.2:c.4197+7C>T	p.? (splice variant)	rs148883310	Damaging[b]	Damaging[b]	0.0194	0.024357	0.028340	2	sALS, fALS	1

Abbreviations: dbSNP = Single Nucleotide Polymorphism database; gnomAD = Genome Aggregation Database; MAF = minor allele frequency; NA = not available

[a] Data from Trans-Omics for Precision Medicine (TopMed) Program
[b] Splice variants were automatically considered deleterious

Table S1. Rare nonsynonymous single nucleotide and splice-site variants found in controls or in ALS patients as well as controls.
